# Supplementary material for: Conformational Effects of Pt-Shells on Nanostructures and Corresponding Oxygen Reduction Reaction Activity of Au-Cluster-Decorated NiOx@Pt Nanocatalysts
Source: Nanomaterials (Basel). 2019 Jul 11;9(7):1003. doi: 10.3390/nano9071003 (PMC6669587; doi:10.3390/nano9071003)
Supplement: Supplementary file 1 [file nanomaterials-09-01003-s001.pdf]

Conformational Effects of Pt-Shells on Nanostructures and Corresponding Oxygen Reduction Reaction Activity of Au-Cluster-Decorated NiO$_x$@Pt Nanocatalysts

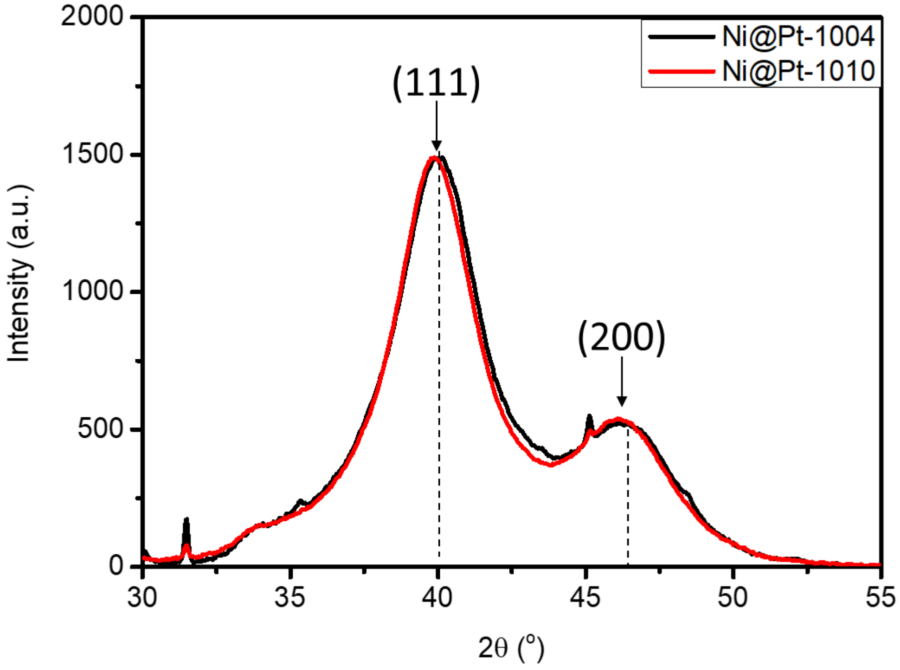

Figure S1. XRD patterns of Ni@Pt-1004 and Ni@Pt-1010 experimental NCs.

Table S1. XRD and TEM determined structural parameters of control samples.

Samples	$Pt_{(111)\ Facet}$		$Pt_{(200)\ facet}$		$D_{(111)}/D_{(200)}$
	d (Å)	D (nm)	d (Å)	D (nm)	
Pt-CNT	2.267	3.52	1.969	2.63	1.34
Ni@Pt-1004	2.239	2.64	1.955	2.38	1.11
Ni@Pt-1010	2.252	2.74	1.965	2.86	0.96

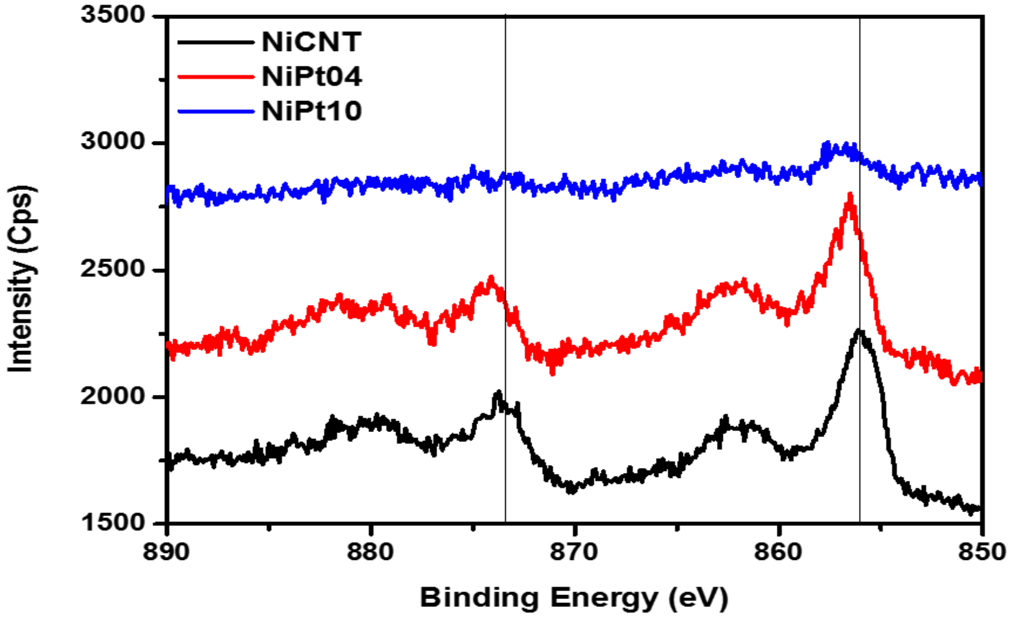

Figure S2. Comparative x-ray photoemission spectroscopy of Ni@Pt-1004 and Ni@Pt-1010 NCs at **Ni**-2p orbitals.

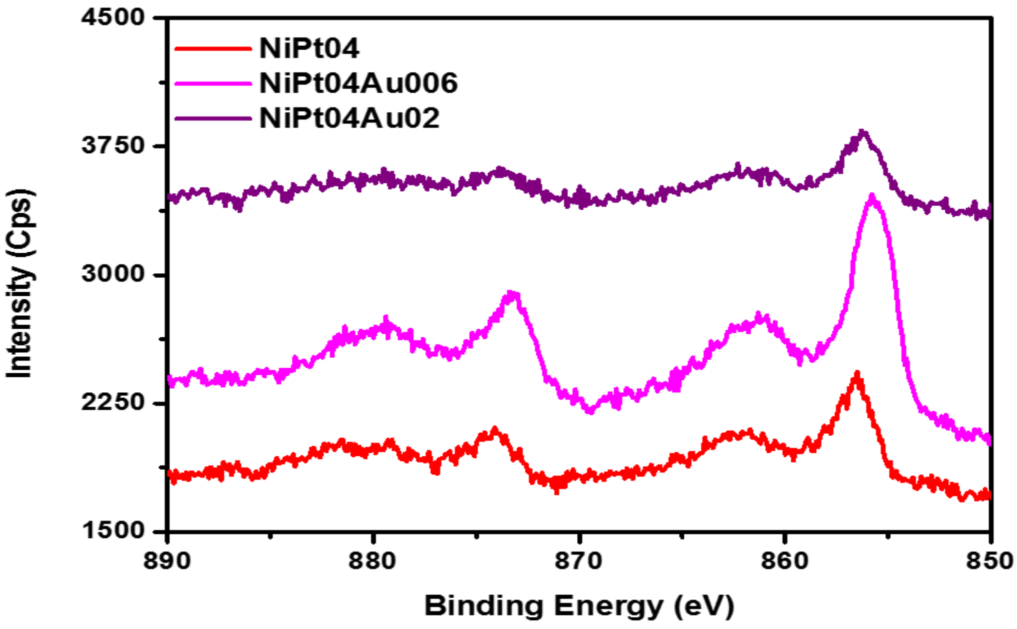

Figure S3. X-ray photoemission spectroscopy of Ni@Pt-1004, NPA-1004006 and NPA-100402 NCs at Ni-2p orbitals of

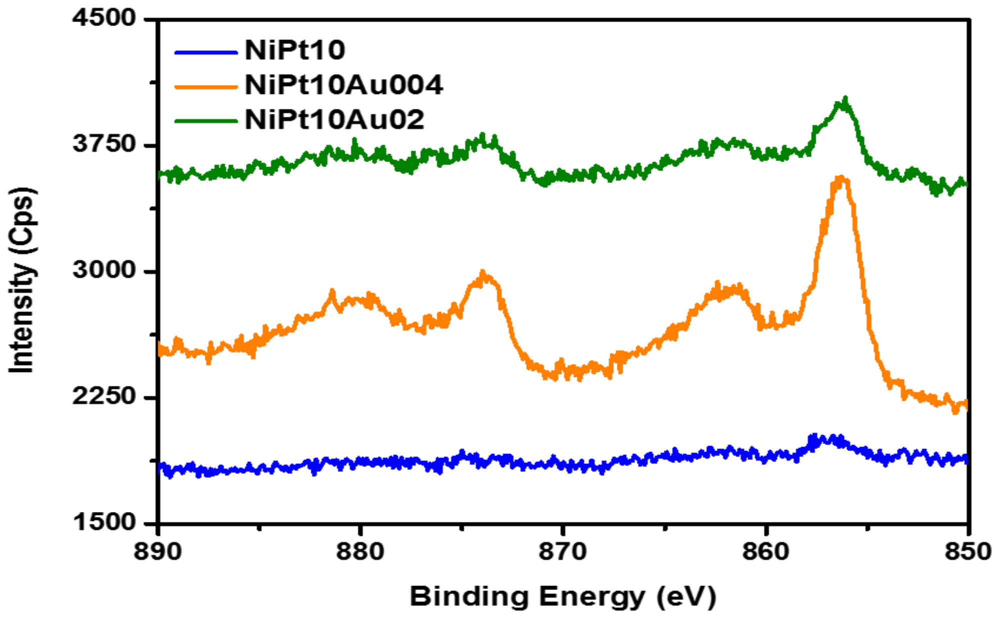

Figure S4. X-ray photoemission spectroscopy of Ni@Pt-1010, NPA-1010006 and NPA-101002 NCs at Ni-2p orbitals.

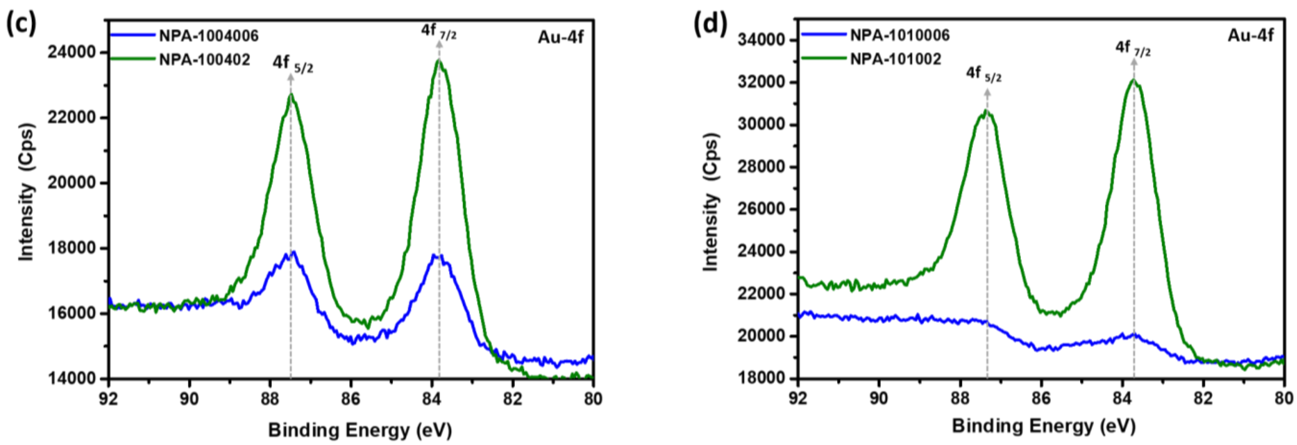

Figure S5. X-ray photoemission spectroscopy of experimental NCs. (a) Au-4f orbitals of NPA-1004006 and NPA-100402. (b) Au-4f orbitals of NPA-1010006 and NPA-101002.

Table S2. Electrochemical parameters of experimental NCs with Pt/Ni = 0.4.

Sample	N(0.5V)	V_{oc} V vs RHE	$E_{1/2}$ V vs RHE	ECSA cm^2mg^{-1}	SA ($mAcm^{-2}$)	JK $_{0.85V}$ $mAcm^{-2}$	M.A. $_{0.85V}$ ($mAmg_{Pt}^{-1}$)
J.M.-Pt/C	4.0	0.910	0.844	257	0.261	4.37	67.0
Ni@Pt-1004	3.3	0.990	0.892	81.2	1.05	9.72	85.2
NPA-1004006	3.5	0.964	0.900	50.0	13.89	75.02	694.5
NPA-100402	3.3	0.969	0.882	49.3	1.83	9.4	90.0

Table S3. Electrochemical parameters of experimental NCs with Pt/Ni = 1.0.

Sample	N(0.5V)	V_{oc} V vs RHE	$E_{1/2}$ V vs RHE	ECSA cm^2mg^{-1}	SA ($mAcm^{-2}$)	JK $_{0.85V}$ $mAcm^{-2}$	M.A. $_{0.85V}$ ($mAmg_{Pt}^{-1}$)
J.M.-Pt/C	4.0	0.910	0.844	257	0.261	4.37	67.0
Ni@Pt-1010	3.8	1.0	0.896	73.1	0.79	12.00	58.0
NPA-1010006	3.7	0.984	0.920	64.4	5.64	73.78	362.9
NPA-101002	3.5	1.01	0.911	71.7	1.61	21.99	115.7

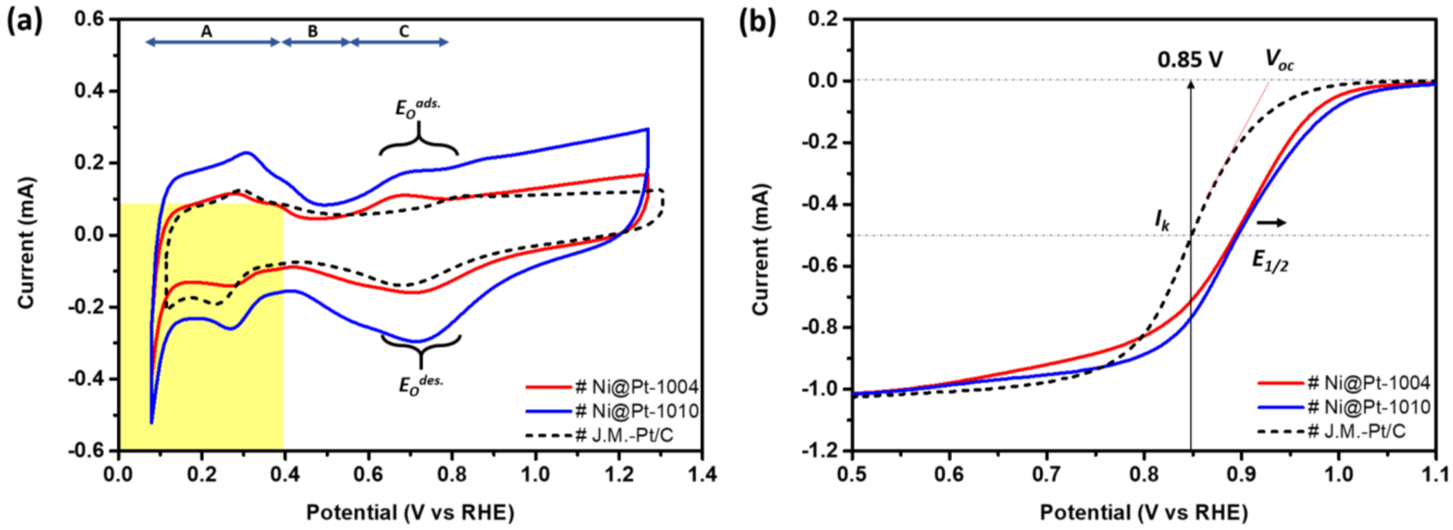

Figure S6. Electrochemical analysis of experimental NCs. (a) CV and (2) LSV curves of Ni@Pt-1004 and Ni@Pt-1010 experimental NCs compared with commercial J.M.-Pt/C nanocatalysts.

Table S4. Electrochemical parameters of control samples.

Sample	N(0.5V)	V_{oc} V vs RHE	$E_{1/2}$ V vs RHE	ECSA cm^2mg^{-1}	SA ($mAcm^{-2}$)	JK $_{0.85V}$ $mAcm^{-2}$	M.A. $_{0.85V}$ ($mAmg_{Pt}^{-1}$)
J.M.-Pt/C	4.0	0.910	0.844	257	0.261	4.37	67.0
Ni@Pt-1004	3.3	0.990	0.892	81.2	1.05	9.72	85.18
Ni@Pt-1010	3.8	1.0	0.896	73.1	0.79	12.00	57.98

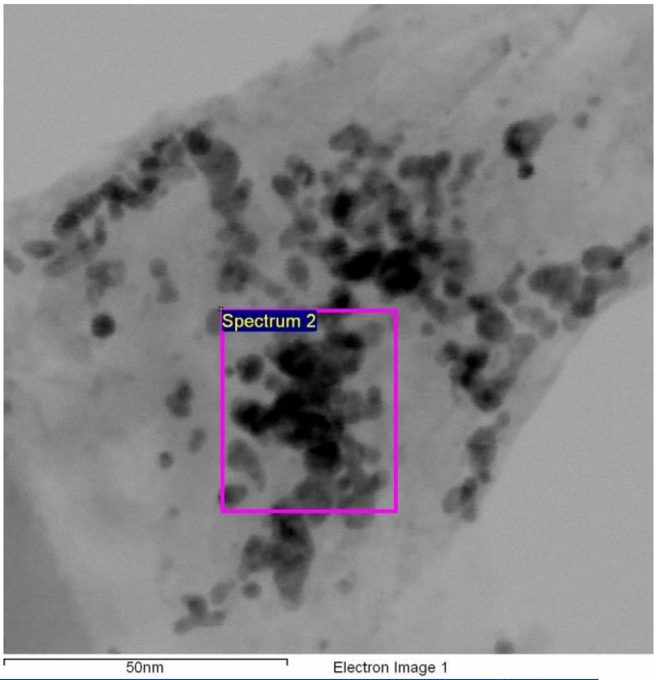

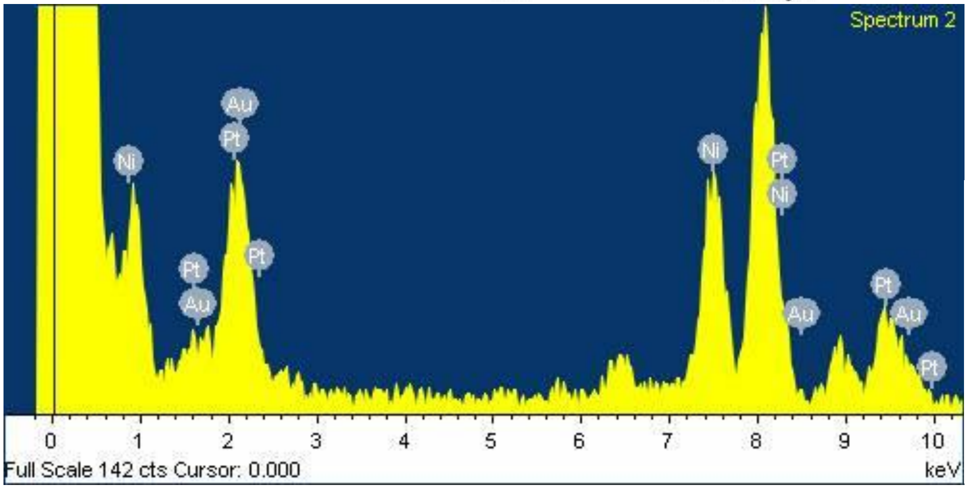

Element	Weight%	Atomic%
Ni K	34.48	63.68
Pt L	47.35	26.32
Au L	18.18	10.01
Totals	100.00	

Figure S7. TEM images of NPA-1004006 NC. The EDX results are measured in the region marked by pink square.
